# Changes in human tear metabolome following topical 0.05% cyclosporine A on primary Sjögren’s syndrome

**DOI:** 10.3389/fmed.2025.1653585

**Published:** 2025-10-24

**Authors:** Yingsi Li, Meiting Huang, Luoying Xie, Xiaoming Yan, Wenjing Song

**Affiliations:** Department of Ophthalmology, Peking University First Hospital, Peking University, Beijing, China

**Keywords:** Sjögren’s syndrome, dry eye disease, cyclosporine A, metabolome, anti-inflammation

## Abstract

**Objective:**

This study aimed to investigate changes in the tear metabolome and the therapeutic impact of 0.05% cyclosporine A (CsA) eye drops in patients with dry eye disease (DED) linked to primary Sjögren’s syndrome (pSS).

**Methods:**

Fifteen patients with pSS-related DED were treated with topical 0.05% CsA for 3 months. Ocular examinations were performed, and tear samples were collected at baseline (T0) and 3 months post-treatment (T1). Differentially expressed metabolites were detected and correlated with clinical parameters.

**Results:**

Topical 0.05% CsA treatment significantly improved the Ocular Surface Disease Index score, lid margin vascularity, conjunctival staining, tear breakup time (TBUT), and lid wiper epitheliopathy (LWE) in DED patients (all *p* < 0.05). A total of 402 metabolites were identified in pSS patients’ tear fluid, with 64 showing differential expression. Pathway analysis identified significant enrichment in the biosynthesis pathways of phenylalanine, tyrosine, and tryptophan. Additionally, certain metabolites (e.g., lipids and anti-inflammatory molecules) correlated positively or negatively with clinical parameters such as TBUT, LWE, and conjunctival staining.

**Conclusion:**

This study underscores significant alterations in tear metabolites at the ocular surface in pSS patients receiving 0.05% topical CsA, offering important insights for managing pSS-related DED clinically.

## 1 Introduction

Dry eye disease (DED) is a chronic condition with multifactorial etiologies that affects millions globally (1, 2). Sjögren’s syndrome (SS) is an autoimmune disease that affects the lacrimal and salivary glands, significantly contributing to aqueous-deficient dry eye and resulting in severe ocular surface damage and visual impairment (3). Whether primary or secondary, the majority of SS cases exhibit meibomian gland dysfunction (MGD), characterized by palpebral margin epithelial metaplasia, obstruction of meibomian gland (MG) orifices, and deficiencies in meibum secretion (4). Inflammation plays a pivotal etiological role in the pathophysiology of DED (2). Chronic ocular surface inflammation increases pro-inflammatory cytokine release, leading to corneal and conjunctival tissue damage (5). Consequently, anti-inflammatory strategies are essential for the treatment of SS-related DED, alongside addressing other contributing factors.

Cyclosporine A (CsA), a common immunomodulator, effectively treats DED and other ocular surface diseases by preventing T-cell infiltration, activation, and proliferation and by inhibiting the transcription of inflammatory cytokine genes such as interleukin-2 and interleukin-4 (6–8). Its mechanism involves forming a complex with cyclophilin to inhibit calcineurin activity, thereby preventing the nuclear translocation and activation of transcription factors in activated T cells (6, 7). The application of topical CsA enhances tear production, preserves ocular surface integrity through its anti-apoptotic and immunomodulatory properties, and mitigates ocular discomfort associated with DED (9–11). Tears, complex fluids containing proteins, lipids, electrolytes, and metabolites, are crucial for identifying biomarkers of ocular surface disorders. Metabolomics, an emerging analytical approach, offers the potential to identify biomarkers and elucidate disease pathways that are more closely aligned with biological phenotypes compared to proteomics. In ophthalmology, metabolomics has contributed significantly to identifying key biomarkers and pathways implicated in the pathogenesis of DED (12). Recent investigations have focused on proteomic alterations in tears following topical CsA administration to pinpoint specific biomarkers (13, 14). Nonetheless, there is a paucity of research examining the changes in tear metabolite levels post-topical CsA treatment. This study aims to investigate and compare the alterations in the tear metabolomic profiles of patients with primary SS (pSS) and DED after treatment with 0.05% topical CsA, with the goal of identifying potential biomarkers.

## 2 Materials and methods

### 2.1 Study design and ethics

This prospective, self-controlled trial was conducted at the Ophthalmology Department of Peking University First Hospital, following approval from the hospital’s Ethics Committee (No. 2021-320). The study complied with the Declaration of Helsinki principles for research involving human subjects. All participants provided written informed consent.

### 2.2 Patient enrollment

Adult participants of any sex with binocular dry eye for a minimum of 6 months, confirmed to have pSS diagnosis from the rheumatology department (15), were recruited. Participants qualified for inclusion if they had a Schirmer I test (SIT) result below 10 mm, a Standard Patient Evaluation of Eye Dryness (SPEED) questionnaire score of at least 6, and a meibomian gland yield secretion score (MGYSS) of 12 or lower for 15 glands per eyelid. The exclusion criteria included any prior ocular surgery, trauma, or infection; eyelid disease; allergies within 6 months before enrollment; punctal plug use; other autoimmune disease-associated DED; severe systemic illnesses; pregnancy or lactation; and contact lens use or alternative treatments (in addition to artificial tears) within 1 month before enrollment. Subjects were administered 0.05% CsA eye drops (Zirun; Shenyang Xingqi Pharmaceutical Co., Ltd., China) four times per day, along with the adjunctive use of artificial tears. Ocular parameters were assessed at the initial time point (T0) and after 3 months (T1) of using CsA treatment.

### 2.3 Clinical assessment

Each participant underwent a comprehensive ophthalmic evaluation for MGD and DED. Subjective symptoms and severity were initially assessed using validated questionnaires, such as the Ocular Surface Disease Index (OSDI) (16) and SPEED questionnaires (17). Lid margin vascularity was evaluated using slit-lamp microscopy on a 0–3 scale, taking into account redness and telangiectasia distribution across the MG orifices, as previously described (18). An MG evaluator applied a consistent pressure of approximately 20 kPa to 15 MG orifices of each eyelid across the temporal, central, and nasal areas for 10 s. Lipid secretion from each gland was evaluated qualitatively using a scale ranging from 0 (no secretion) to 3 (clear liquid secretion) (19). The analysis involved calculating total scores for vascularity (0 to 6) and MGYSS (0 to 90) for the upper and lower eyelids of one eye. Fluorescein sodium and lissamine green strips were used for ocular surface staining. Corneal fluorescein staining (CFS) was assessed with the National Eye Institute/Industry scale (20), and conjunctival staining was evaluated using the Oxford Scheme (21). Lid wiper epitheliopathy (LWE) was assessed with lissamine green staining, and scores were averaged based on horizontal staining (0–3) and sagittal height (0–3) of the lid wiper (22). The total upper and lower LWE scores were combined, resulting in a scale from 0 to 6. Tear film stability and secretion were assessed by measuring the average tear breakup time (TBUT) and Schirmer I test (SIT), respectively. Evaluations were performed separately for the right and left eyes.

### 2.4 Tear sample collection

Tear samples were obtained from both eyes at two time points, T0 and T1, following strict standardized procedures and times. Briefly, a sterile microtube was used to administer 100 μL of sterile saline into the conjunctival sac of the lower eyelid. Patients were instructed to move their eyeballs up, down, left, and right four times without blinking to ensure thorough mixing of the saline with the tear fluid. Tear samples were immediately collected with a micropipette, stored in Eppendorf tubes, and promptly frozen at −80 °C. Tears from one eye per patient were randomly chosen for non-targeted metabolomics analysis, which was conducted by Shanghai Genechem Co., Ltd. (Shanghai, China).

### 2.5 Liquid chromatography-tandem mass spectrometry analysis

Tear samples were thawed and dissolved at 4 °C, and then 100 μL was transferred to a sterile Eppendorf tube and vortexed with pre-chilled 80% methanol. The mixed samples were incubated on ice for 5 min and then centrifuged at 15,000 g and 4 °C for 20 min. The supernatant was diluted with LC-MS grade water to achieve a 53% methanol concentration and then centrifuged again. The final supernatant was injected into a Hypersil Gold column (100 × 2.1 mm, 1.9 μm; Thermo Fisher Scientific, Germany) using a 12-min linear gradient at a flow rate of 0.2 mL/min. Quality control (QC) samples were created by mixing 50 μL from each sample, while a blank sample was prepared with a 53% methanol-aqueous solution. Liquid chromatography-tandem mass spectrometry (LC-MS/MS) data were obtained using a Vanquish UHPLC system paired with an Orbitrap Q Exactive^™^ HF-X mass spectrometer, both from Thermo Fisher Scientific, Germany. The initial three QC samples, analyzed prior to sample injection, were used to assess instrument performance and stabilize the LC-MS/MS system. Segmented scanning and secondary spectral analysis of experimental samples were conducted to identify metabolites. The final supernatant was then injected into a Hypersil Gold column (Thermo Fisher Scientific, Germany), with the mobile phase composition and gradient settings following a previously published method in reference (23). Configured in full scan mode, the Q ExactiveTM HF mass spectrometer operated with a sheath gas flow rate of 35 psi, a spray voltage of 3.5 kV, a capillary temperature of 320 °C, and an auxiliary gas flow rate of 10 L/min, with an S-lens RF level of 60 and auxiliary gas heating at 350 °C.

### 2.6 Data processing and metabolite identification

Compound Discoverer (version 3.3) was used to process the raw data for metabolite peak alignment, peak picking, and quantitation. Key parameters were set as previously reported (24). Normalized data were used to predict molecular formulas based on additive ions, molecular ion peaks, and fragment ions. Subsequently, peaks were verified using the mzCloud, mzVault, and MassList databases to ensure accuracy. The normalized peak data underwent additional preprocessing using metaX, applying the formula: sample raw quantitation value divided by the ratio of the sum of sample metabolite quantitation values to the sum of QC1 sample metabolite quantitation values. Compounds with a QC variance exceeding 30% were removed after standardizing the data to obtain relative peak area values. Principal component analysis (PCA) and partial least squares discriminant analysis (PLS-DA) were applied as multivariate statistical methods. A volcano plot was drawn by R (version 3.4.3) with the ggplot2 package. Tear metabolites were considered differentially expressed if they had a variable importance in projection (VIP) score above 1, a *p*-value below 0.05 from a Student’s t-test, and a fold change (FC) exceeding 1.5 or below 0.67. These differential metabolites were annotated in the KEGG database,^1^ the LIPID MAPS database,^2^ and the Human Metabolome Database (HMDB).^3^ KEGG enrichment analysis was performed at https://new.metaboanalyst.ca, and clustering heat maps were generated using the pheatmap package based on *Z*-scores of differential metabolite intensity areas. Spearman’s correlation analysis was conducted using SPSS 18.0 (IBM Corp., Armonk, NY, United States) to evaluate the correlation coefficients between clinical data and tear fluid metabolites.

### 2.7 Statistical analysis

Paired t-tests were used to assess differences in ocular parameters between pre- and post-treatment samples that followed a normal distribution, with results expressed as mean ± standard deviation. Clinical parameter analyses were conducted using SPSS 18.0, with statistical significance set at a two-tailed *p*-value of <0.05.

## 3 Results

### 3.1 Ocular surface alterations in pSS patients

Our study included 15 participants, comprising 1 man and 14 women, with a mean age of 55 years (SD: 10.76) and an age range of 37–72 years. Among the enrolled subjects, the ocular surface changes in pSS patients with DED are detailed in Table 1. After treatment with topical 0.05% CsA, the OSDI questionnaire score significantly decreased from 12.60 ± 6.93 to 8.20 ± 6.24 (*p* = 0.025). However, the SPEED score remained unaffected. Compared with baseline, TBUT markedly increased for both eyes (*p* = 0.005 and *p* = 0.003), whereas conjunctival staining significantly decreased (*p* = 0.006 and *p* = 0.004, respectively). Additionally, the vascularity of the lid margin improved significantly in both eyes (*p* = 0.027 and *p* = 0.028, respectively). The LWE score in the left eye also significantly decreased (*p* = 0.003), although no significant change was observed in the right eye. No significant differences were observed in the MGYSS, CFS, or SIT.

**Table 1:** Ocular surface alterations of pSS patients with 0.05% CsA treatment (*n* = 15).

	OD			OS		
Parameters	T0	T1	*p*-value	T0	T1	*p*-value
Vascularity of lid margin	2.67 ± 0.82	2.00 ± 1.36	**0.027** ^*^	2.67 ± 0.82	1.87 ± 1.30	**0.028** ^*^
MGYSS	5.07 ± 6.89	6.67 ± 5.93	0.510	5.64 ± 9.03	6.08 ± 7.50	0.801
LWE	1.67 ± 1.80	0.71 ± 1.25	0.289	2.22 ± 1.09	0.42 ± 1.13	**0.003** ^**^
SIT	4.64 ± 4.95	6.83 ± 7.94	0.436	4.73 ± 5.57	6.42 ± 4.76	0.248
CFS	5.27 ± 3.94	3.07 ± 4.38	0.104	4.13 ± 4.05	2.73 ± 2.96	0.309
Conjunctival staining	3.73 ± 2.94	1.00 ± 2.17	**0.006** ^**^	3.87 ± 3.16	0.67 ± 1.18	**0.004** ^**^
TBUT	1.82 ± 0.57	2.71 ± 1.29	**0.005** ^**^	1.74 ± 0.57	2.84 ± 1.24	**0.003** ^**^
SPEED	7.93 ± 4.46	6.53 ± 4.07	0.290	7.53 ± 4.26	7.14 ± 3.35	0.471
OSDI	12.60 ± 6.93	8.20 ± 6.24	**0.025** ^*^	12.60 ± 6.93	8.20 ± 6.24	**0.025** ^*^

A paired *t*-test was conducted for statistical analysis, with data expressed as mean ± standard deviation. ^*^p < 0.05 and ^**^p < 0.01. MGYSS, meibomian gland yield secretion score; LWE, lid wiper epitheliopathy; SIT, Schirmer I test; CFS, corneal fluorescein staining; TBUT, tear breakup time; SPEED, Standard Patient Evaluation of Eye Dryness; OSDI, ocular surface disease index. The bold values in this table indicate *p* < 0.05 and are highlighted to emphasize statistically significant findings.

### 3.2 Metabolic variations in pSS patients following CsA therapy

**Figure 1:**
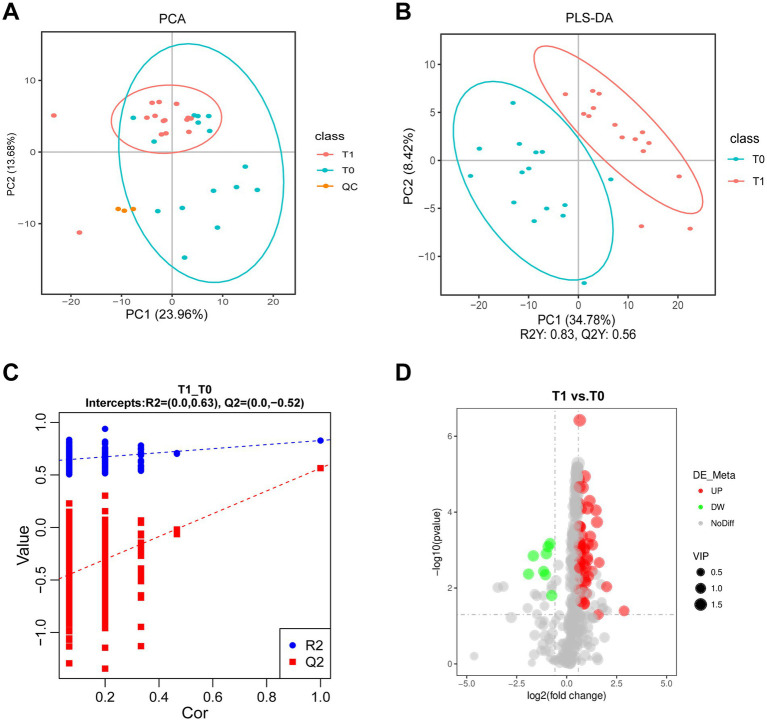
Identified metabolites in tear fluid from pSS patients with 0.05% CsA treatment. **(A)** PCA diagram of the T0 and T1 groups. **(B,C)** PLS-DA and permutation plots among the T0 and T1 groups. *R*^2^*Y* indicates the model’s interpretation rate, while *Q*^2^*Y* assesses the predictive capability of the PLS-DA model, with values of *R*^2^*Y* = 0.83 and *Q*^2^*Y* = 0.56. The model demonstrates strong performance as indicated by *R*^2^*Y* being greater than *Q*^2^*Y* and the Q2 regression line’s intercept with the *Y*-axis being less than zero. **(D)** Volcano plot illustrates metabolite differences between the T0 and T1 groups.

Untargeted metabolomics was performed on tear samples from 15 pSS patients. A multivariate statistical analysis using PCA and PLS-DA models demonstrated a clear separation between tear samples before and after CsA treatment (Figures 1A–C), which confirmed the reliability of the model. QC points clustered tightly in the center of the scores plot indicated stable metabolomics platform performance and high data quality, as further demonstrated by QC sample correlation (Supplementary Figure S1). Through LC-MS/MS spectral analysis, a total of 402 metabolites and 64 differentially abundant metabolites between T0 and T1 were identified. Specifically, 8 metabolites were downregulated and 52 were upregulated following 0.05% CsA treatment, as shown in the volcano plot (Figure 1D). The relative contents of all differentially abundant metabolites in each sample are provided in Supplementary Table S1.

### 3.3 Metabolic pathways in pSS patients with CsA therapy

**Figure 2:**
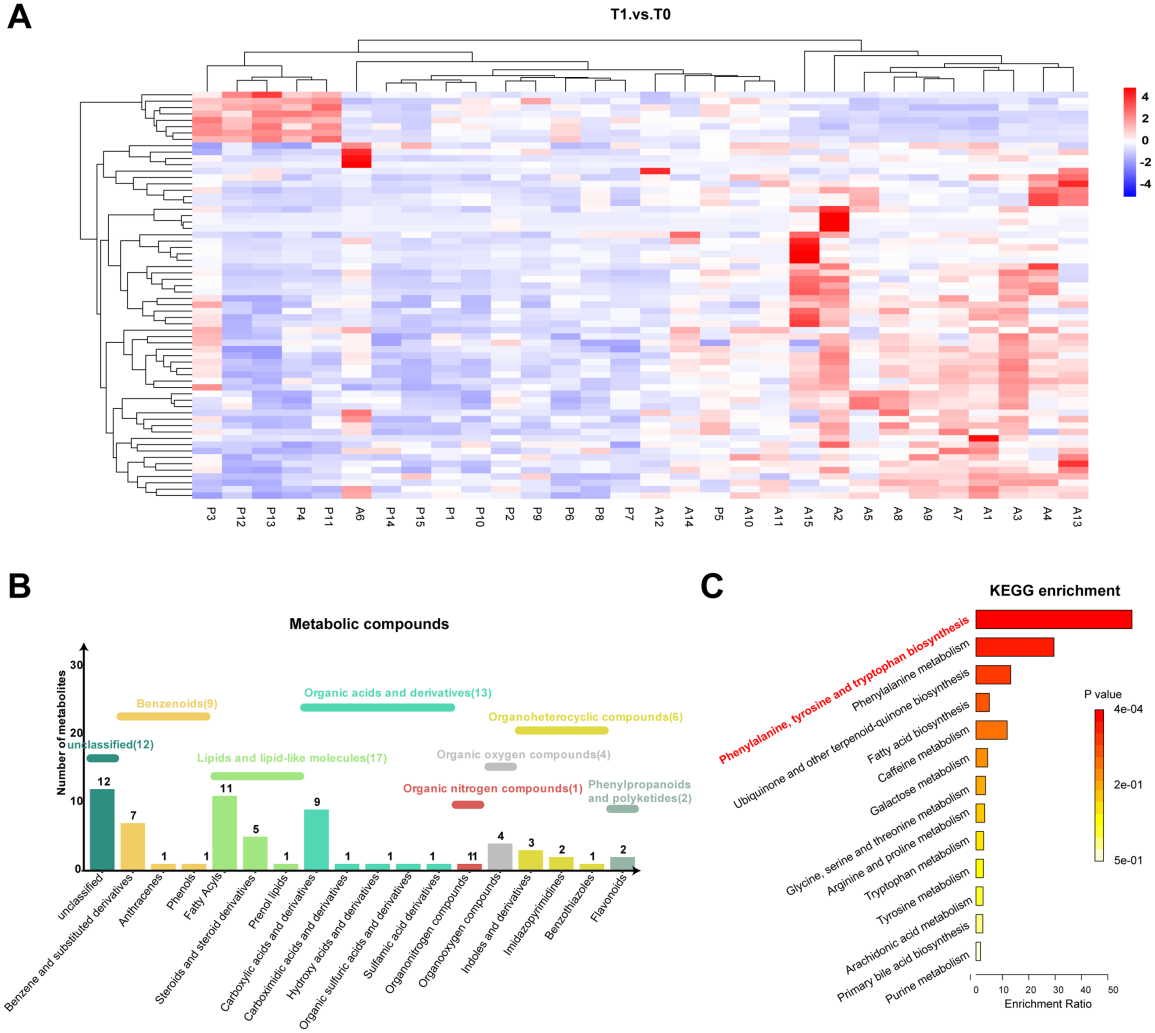
Metabonomic pathway of significantly different metabolites. **(A)** Heatmap of tear differentially expressed metabolites among both groups. **(B,C)** Category histogram and KEGG enrichment analysis of differentially expressed metabolites in tears.

As described above, significant alterations in the metabonomic profiles of tears from pSS patients treated with topical 0.05% CsA were observed. As shown in Figure 2A, the heatmap shows the variations in metabolic molecules in all tear samples. The identified differential tear metabolites were divided into seven categories according to HMDB, among which the most diverse were lipids and lipid-like molecules (*n* = 17), followed by organic acids and derivatives (*n* = 13) and benzenoids (*n* = 9) (Figure 2B). Consistent with prior studies, the predominant lipids were fatty acyls, steroids, and steroid derivatives (25). KEGG pathway analysis identified significant enrichment in the biosynthesis of phenylalanine, tyrosine, and tryptophan pathways (Figure 2C). Supplementary Table S1 provides the detailed metabolic pathways/categories and putative biological roles from KEGG, HMDB, and LIPID MAPS public resources.

### 3.4 Correlation between metabolites and ocular surface parameters

**Figure 3:**
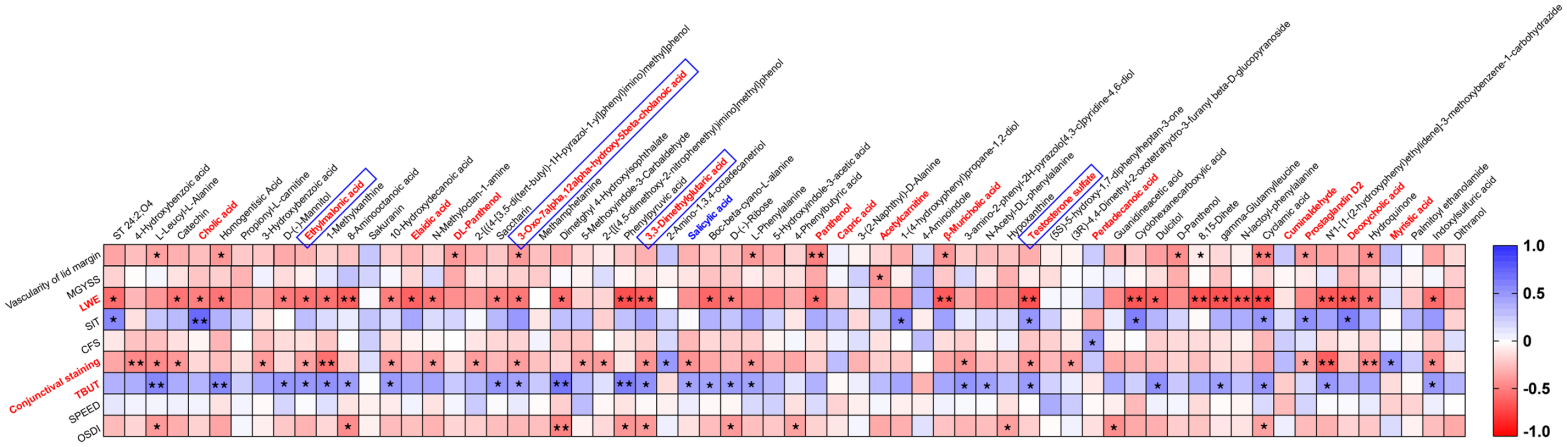
Clustered Spearman’s correlation matrix for clinical parameters and 64 metabolites. Spearman’s rank correlation coefficient was used to calculate correlation coefficients (*r*) and *p*-values. The color key shows the correlation coefficient (*r*), with asterisks indicating *p*-values (^*^p < 0.05 and ^**^p < 0.01) in the cells.

Subsequent correlation analysis revealed significant moderate correlations between the tear metabolites and clinical parameters (Figure 3). Many metabolites exhibited positive and negative correlations with TBUT, LWE, and conjunctival staining, with LWE showing particularly strong correlations. Lipid molecules are crucial for tear film stability and evaporation prevention (26). Of the 17 lipids and lipid-like molecules analyzed, four metabolites showed a positive correlation with TBUT. The compounds 3,3-dimethylglutaric acid (*r* = 0.428) and ethylmalonic acid (*r* = 0.407) belong to the fatty acyl group, while 3-oxo-7alpha,12alpha-hydroxy-5beta-cholanoic acid (*r* = 0.363) and testosterone sulfate (*r* = 0.392) are categorized as steroids and their derivatives. Moreover, the levels of anti-inflammatory prostaglandin D2 (PGD2) were negatively related to the vascularity of the lid margin and conjunctival staining and positively associated with the SIT. Additionally, the presence of salicylic acid, another non-steroidal anti-inflammatory molecule present in tears, was related to TBUT and conjunctival staining.

## 4 Discussion

Over the past two decades, CsA has been a well-established immunomodulator with remarkable efficacy in treating ocular surface diseases. Research has demonstrated that it can increase tear production, stabilize the tear film, reduce keratoconjunctival epithelial cell apoptosis, and suppress both inflammatory cell activation and cytokine expression on the ocular surface (7, 11). This study explored functional improvement and biochemical alterations in pSS-associated DED patients treated with 0.05% CsA by combining validated clinical assessments with untargeted metabolomics.

Consistent with previous studies, we revealed that topical 0.05% CsA treatment could ameliorate ocular discomfort and reduce eyelid congestion and conjunctival staining scores (9, 27). An unexpected finding was the reduction in the grade of LWE following topical 0.05% CsA treatment, a phenomenon not previously observed with CsA treatment. However, this effect was observed only in the left eye in the current study, and expanding the sample size is likely to yield more stable results. LWE, an epitheliopathy of the eyelid’s marginal conjunctiva, is strongly linked to DED, MGD, and contact lens use (28, 29). Our recent study showed that LWE could serve as an initial sign for DED diagnosis (30). LWE indicates the areas of damaged conjunctival goblet cells, leading to insufficient ocular lubrication or increased frictional interaction (31). Our decreased LWE scores suggested the potential of CsA to improve the function of conjunctival epithelial cells, thereby enhancing the tear film and reducing abnormal frictional forces, which may halt the pathophysiology of the condition. These findings were consistent with the increase in conjunctival goblet cell density and the inhibition of conjunctival goblet cell apoptosis (7, 11). Furthermore, correlation analysis revealed that LWE was closely related to the differentially expressed metabolites, which correlated well with 31 metabolites. Notably, the observed asymmetry of LWE should be interpreted with caution due to the limited sample size, and future larger-scale studies are needed to confirm the consistency of the effect across both eyes. These results underscore the significance of LWE as an observational indicator of pSS-associated DED and its potential role in treatment monitoring.

In addition to the functional and pathological changes in the lacrimal gland, pSS patients typically experience MGD (32). This study included pSS patients with both DED and MGD. Previous evidence has suggested that T cells have a minor contribution to the development and progression of MG, resulting in a weaker response to CsA (11). Consequently, CsA treatment could not completely reverse all the symptoms and signs, such as MG loss and meibum quality scores, which were consistent with previous literature (33, 34). The participants showed a significant increase in TBUT in both eyes after 3 months of CsA treatment, indicating improved tear film stability and a reduced tear evaporation rate. MG is the main contributor to lipid secretion on the ocular surface. Abnormal gland function can lead to the dysfunction of the composition and quality of the meibum. Through metabolomics analysis, we found that a panel of tear metabolites was altered by CsA treatment. Among these, we confirmed that CsA improved 17 lipids and lipid-like molecules, with 4 lipids positively correlated with TBUT. However, these molecules have been detected in tears for the first time, and their functions and regulatory mechanisms still require further investigation. According to a previous study (35), there can be as many as 323 lipid molecules in extruded meibum, but we detected only 92 lipid molecules with untargeted metabolomics. Utilizing lipidomics and targeted metabolomics to detect extruded meibum may yield additional insights for future analyses.

CsA is a cyclic polypeptide with potent immunosuppressive properties. Our research revealed that CsA can increase the concentration of PGD2, a lipid mediator with diverse biological functions, in tears. In contrast to the proinflammatory effects of prostaglandin E2, PGD2 demonstrated anti-inflammatory properties and offered protection against inflammatory damage. According to prior findings by Shim et al. (36), compared with healthy subjects, patients with non-pSS-type-DED exhibited decreased PGD2 levels in their tears, which were related to their irritation scores. In the present research, we demonstrated that PGD2 levels were negatively related to the vascularity of the lid margin and conjunctival staining and positively related to the SIT. The elevated PGD2 levels in tears following the use of topical 0.05% CsA further reinforced its anti-inflammatory effects, whereas the correlation analysis between PGD2 and DED questionnaires revealed no statistical significance. Additionally, we found that cyclosporine could increase the activity of salicylic acid, another non-steroidal anti-inflammatory molecule. Previously, 0.1% salicylic acid eye drops were shown to effectively treat eyelid conjunctivitis by significantly enhancing ocular surface inflammation and tear film stability (37). These findings confirm the anti-inflammatory effect of CsA and further suggest that PGD2 and salicylic acid in tears could be used as markers for DED.

Apart from the alteration of lipid metabolism and anti-inflammatory factors mentioned above, we also found a significant enrichment of the phenylalanine-tyrosine-tryptophan pathway in the tear fluid, with elevated levels of L-phenylalanine and phenylpyruvic acid in pSS-associated DED patients after topical CsA therapy. L-Phenylalanine is an essential amino acid for humans and animals and is oxidized into L-tyrosine by phenylalanine hydroxylase catalysis, after which it is subsequently converted into important neurotransmitters and hormones (38). In an experimental DED model study with Wistar rats, researchers reported decreased levels of L-phenylalanine and phenylpyruvic acid in the central cornea (39), unlike other studies that reported increased levels in the tears of DED patients (40, 41). Furthermore, a separate study revealed that multiple sclerosis patients had lower levels of phenylalanine and tryptophan in their tears than healthy subjects did (42). However, the function and regulatory mechanisms of amino acid metabolism at the ocular surface remain poorly defined. Following CsA therapy, the levels of several tear metabolites increase or decrease in pSS patients; however, whether these changes reflect a direct pharmacodynamic effect of CsA or simply mirror an improved ocular-surface microenvironment remains unclear. By correlating specific metabolites—namely, lipids, anti-inflammatory mediators, and amino acids—with validated clinical indices, we identified candidate biomarkers that may facilitate treatment-response monitoring and guide future therapeutic strategies. However, since non-targeted metabolomics provided relative quantification with limited reproducibility, complementing with targeted or lipidomics analyses could validate and refine biomarker findings in the future.

A limitation of our study is the use of saline instillation in tear collection, which diluted native tear components and could theoretically impact absolute metabolite concentrations and the detection sensitivity for low-abundance metabolites. Techniques for reducing the salt load and micro-sampling technologies to obtain richer, more concentrated metabolomic information from minimal tear volumes are needed in the future. Another shortcoming is the small sample size and the lack of control and non-pSS groups. It would be strengthened by including a larger cohort to improve statistical power and enhance the generalizability of the findings. Adding a healthy control group and non-pSS-related DED subjects would help clarify whether the observed metabolomic changes are specific to pSS or CsA treatment. Moreover, it is important to emphasize the possible impact of using artificial tears as an adjunct on metabolomics data. There are optimized formulations, of which the novel developed formulations, such as nanomicellar solutions, have been shown to increase tolerability, bioavailability, and retention time (8, 43, 44). It would be interesting to further test novel formulations, such as nanomicellar and higher concentrations of CsA, on pSS. Moreover, extending the follow-up period would enable the assessment of the long-term stability of both clinical and metabolomic improvements, providing stronger evidence for therapeutic efficacy.

## 5 Conclusion

Overall, topical CsA treatment has the potential to ameliorate lid margin congestion, conjunctival staining, LWE, and tear film stability in patients with pSS-related DED. Furthermore, it enhanced anti-inflammatory molecules in tears to suppress immune-inflammatory responses, regulated lipid and amino acid metabolism, and ultimately maintained ocular surface health. These findings offer valuable insights for medical approaches targeting pSS-related DED.

## Data Availability

The original contributions presented in the study are included in the article/Supplementary material, further inquiries can be directed to the corresponding authors.

## Footnotes

^1^ https://www.genome.jp/kegg/pathway.html

^2^ https://lipidmaps.org/

^3^ https://www.hmdb.ca/
