# Supplementary material for: Changes in human tear metabolome following topical 0.05% cyclosporine A on primary Sjögren’s syndrome
Source: Front Med (Lausanne). 2025 Oct 24;12:1653585. doi: 10.3389/fmed.2025.1653585 (PMC12592072; doi:10.3389/fmed.2025.1653585)
Supplement: Supplementary file 3 [file Supplementary_file_1.docx]

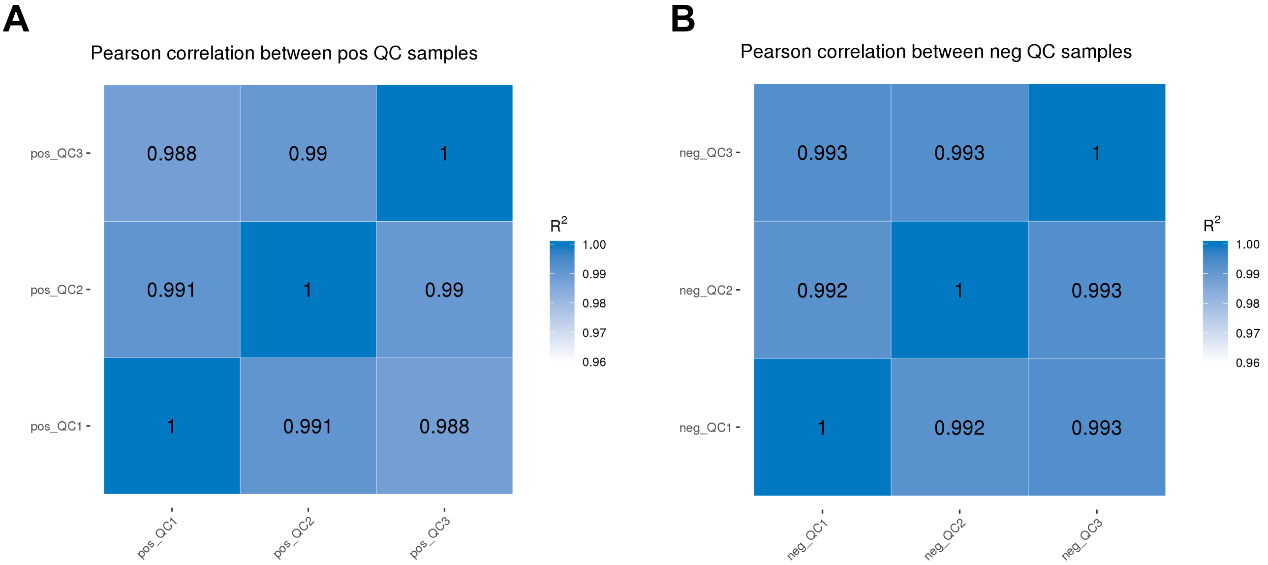


Supplementary Figure S1 QC samples correlation

(A) Positive-mode and (B) negative-mode Pearson correlations of QC samples. Coefficients were computed from the relative abundance of all detected features; R² ≈ 1 confirms stable analytical performance and high data quality.
